# The genome sequence of the Little Grey, *Eudonia lacustrata *(Panzer, 1804)

**DOI:** 10.12688/wellcomeopenres.19646.1

**Published:** 2023-07-11

**Authors:** Douglas Boyes, Liam M. Crowley, Clare Boyes, University of Oxford and Wytham Woods Genome Acquisition Lab, Darwin Tree of Life Barcoding collective, Wellcome Sanger Institute Tree of Life programme, Wellcome Sanger Institute Scientific Operations: DNA Pipelines collective, Tree of Life Core Informatics collective, Darwin Tree of Life Consortium

**Affiliations:** 1 UK Centre for Ecology & Hydrology, Wallingford, England, UK; 2 University of Oxford, Oxford, England, UK; 3 Independent researcher, Welshpool, Wales, UK

**Keywords:** Eudonia lacustrata, Little Grey, genome sequence, chromosomal, Lepidoptera

## Abstract

We present a genome assembly from an individual male *Eudonia lacustrata* (the Little Grey; Arthropoda; Insecta; Lepidoptera; Crambidae). The genome sequence is 699.5 megabases in span. Most of the assembly is scaffolded into 30 chromosomal pseudomolecules, including the Z sex chromosome. The mitochondrial genome has also been assembled and is 15.29 kilobases in length. Gene annotation of this assembly on Ensembl identified 21,652 protein coding genes.

## Species taxonomy

Eukaryota; Metazoa; Eumetazoa; Bilateria; Protostomia; Ecdysozoa; Panarthropoda; Arthropoda; Mandibulata; Pancrustacea; Hexapoda; Insecta; Dicondylia; Pterygota; Neoptera; Endopterygota; Amphiesmenoptera; Lepidoptera; Glossata; Neolepidoptera; Heteroneura; Ditrysia; Obtectomera; Pyraloidea; Crambidae; Scopariinae; *Eudonia*; *Eudonia lacustrata* (Panzer, 1804) (NCBI:txid1100991).

## Background

*Eudonia lacustrata* (Little Grey) is a common micro-moth in the family Crambidae. The moth is common and widespread throughout most of Britain. The species occurs throughout Europe, apart from the far north, and east to western China ( Leraut, 2012)

In the UK, the adult moth is on the wing from mid-May to August and is single-brooded. The moth is found in open habitats, woodland, parks and gardens. *E. lacustrata* is a small moth with a forewing length of 8–9 mm. The light grey forewing has dark speckling, and a pattern of darker grey cross lines, and spots. The adult rests in the day on tree-trunks and walls, and flies at night, when it readily comes to light. The larvae, which are contained within a silk tube, feed on mosses usually growing on tree-trunks and walls ( Sterling & Parsons, 2018).

A genome sequence from *E. lacustrata* will be useful for comparative studies across the Lepidoptera. The genome of *E. lacustrata* was sequenced as part of the Darwin Tree of Life Project, a collaborative effort to sequence all named eukaryotic species in the Atlantic Archipelago of Britain and Ireland. Here we present a chromosomally complete genome sequence for *E. lacustrata* based on male specimen from Wytham Woods, Oxfordshire, UK.

## Genome sequence report

**Figure 1.**
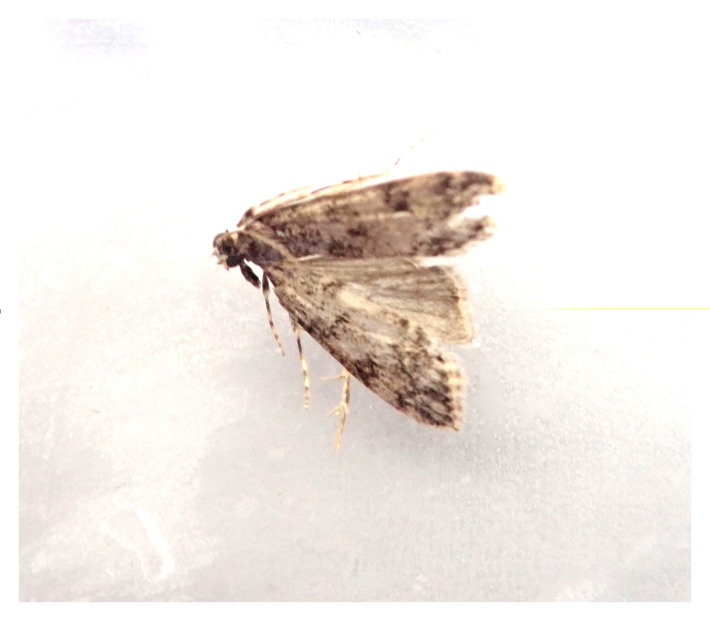
Photograph of the *Eudonia lacustrata* (ilEudLacu3) specimen used for genome sequencing.

The genome was sequenced from one male *Eudonia lacustrata* ( Figure 1) collected from Wytham Woods, Oxfordshire (51.77, –1.34). A total of 36-fold coverage in Pacific Biosciences single-molecule HiFi long reads was generated. Primary assembly contigs were scaffolded with chromosome conformation Hi-C data. Manual assembly curation corrected 30 missing joins or mis-joins and removed 9 haplotypic duplications, reducing the assembly length by 0.26% and the scaffold number by 17.91%, and increasing the scaffold N50 by 0.54%.

**Figure 2.**
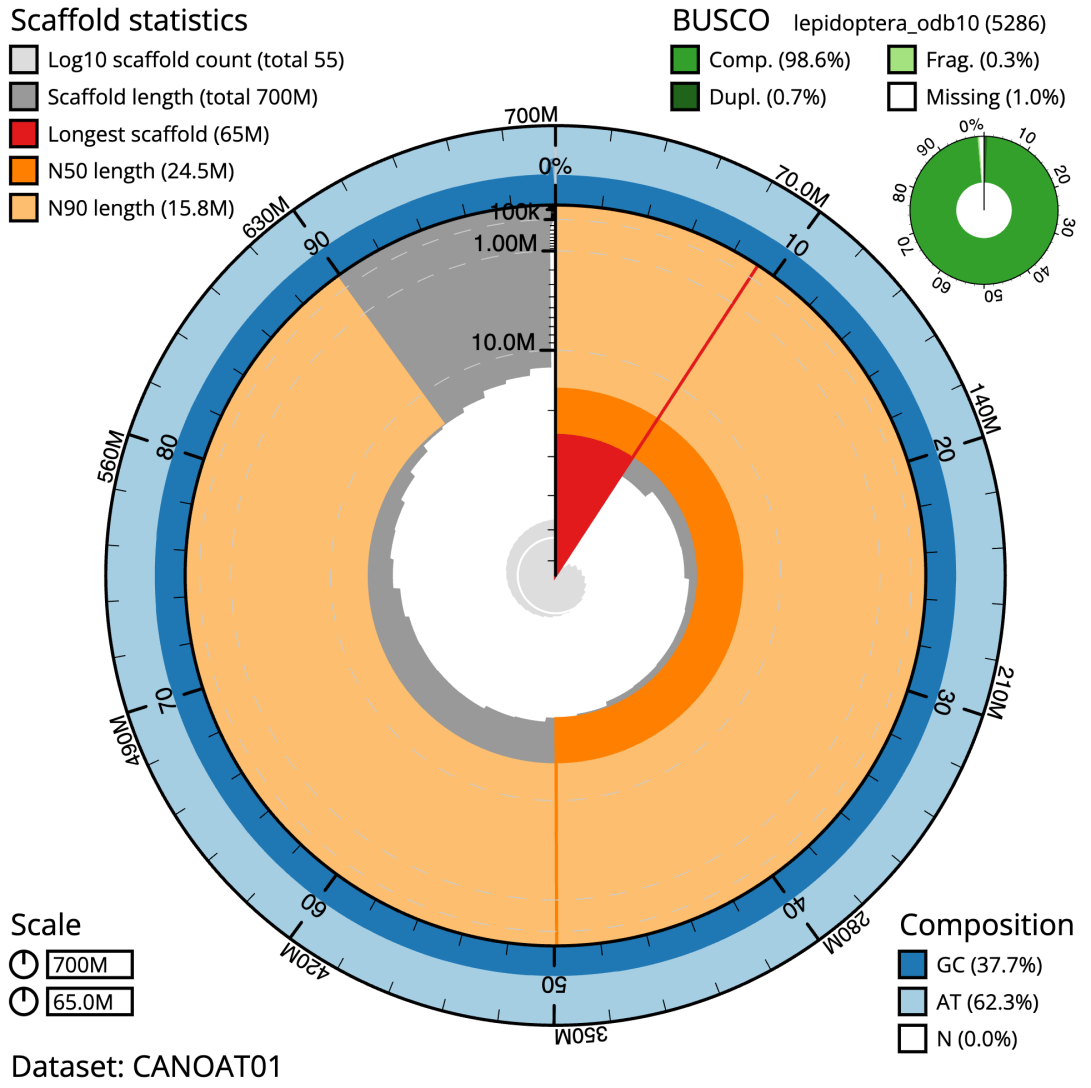
Genome assembly of *Eudonia lacustrata*, ilEudLacu3.1: metrics. The BlobToolKit Snailplot shows N50 metrics and BUSCO gene completeness. The main plot is divided into 1,000 size-ordered bins around the circumference with each bin representing 0.1% of the 699,510,583 bp assembly. The distribution of scaffold lengths is shown in dark grey with the plot radius scaled to the longest scaffold present in the assembly (65,033,386 bp, shown in red). Orange and pale-orange arcs show the N50 and N90 scaffold lengths (24,461,152 and 15,798,145 bp), respectively. The pale grey spiral shows the cumulative scaffold count on a log scale with white scale lines showing successive orders of magnitude. The blue and pale-blue area around the outside of the plot shows the distribution of GC, AT and N percentages in the same bins as the inner plot. A summary of complete, fragmented, duplicated and missing BUSCO genes in the lepidoptera_odb10 set is shown in the top right. An interactive version of this figure is available at https://blobtoolkit.genomehubs.org/view/ilEudLacu3.1/dataset/CANOAT01/snail.

The final assembly has a total length of 699.5 Mb in 54 sequence scaffolds with a scaffold N50 of 24.5 Mb ( Table 1). Most (99.67%) of the assembly sequence was assigned to 30 chromosomal-level scaffolds, representing 29 autosomes and the Z sex chromosome. Chromosome-scale scaffolds confirmed by the Hi-C data are named in order of size ( Figure 2– Figure 5; Table 2). While not fully phased, the assembly deposited is of one haplotype. Contigs corresponding to the second haplotype have also been deposited. The mitochondrial genome was also assembled and can be found as a contig within the multifasta file of the genome submission.

**Table 1.** Genome data for *Eudonia lacustrata*, ilEudLacu3.1.

Project accession data		
Assembly identifier	ilEudLacu3.1	
Species	*Eudonia lacustrata*	
Specimen	ilEudLacu3	
NCBI taxonomy ID	1100991	
BioProject	PRJEB56735	
BioSample ID	SAMEA7701440	
Isolate information	ilEudLacu3, whole organism (DNA sequencing); ilEudLacu4, whole organism (Hi-C), ilEudLacu5; whole organism (RNA sequencing)	
Assembly metrics *		*Benchmark*
Consensus quality (QV)	60.6	*≥ 50*
*k*-mer completeness	100%	*≥ 95%*
BUSCO **	C:98.6%[S:98.0%,D:0.7%], F:0.3%,M:1.0%,n:5,286	*C ≥ 95%*
Percentage of assembly mapped to chromosomes	99,67%	*≥ 95%*
Sex chromosomes	Z chromosome	*localised homologous pairs*
Organelles	Mitochondrial genome assembled	*complete single alleles*
Raw data accessions		
PacificBiosciences SEQUEL II	ERR10395970, ERR10395971	
10X Genomics Illumina	ERR10378041–ERR10378048	
Hi-C Illumina	ERR10378049	
PolyA RNA-Seq Illumina	ERR11242510	
Genome assembly		
Assembly accession	GCA_947562085.1	
*Accession of alternate * *haplotype*	GCA_947562065.1	
Span (Mb)	699.5	
Number of contigs	229	
Contig N50 length (Mb)	5.5	
Number of scaffolds	54	
Scaffold N50 length (Mb)	24.5	
Longest scaffold (Mb)	65.0	
Genome annotation		
Number of protein- coding genes	21,652	
Number of gene transcripts	21,845	

* Assembly metric benchmarks are adapted from column VGP-2020 of “Table 1: Proposed standards and metrics for defining genome assembly quality” from ( Rhie *et al.*, 2021). ** BUSCO scores based on the lepidoptera_odb10 BUSCO set using v5.3.2. C = complete [S = single copy, D = duplicated], F = fragmented, M = missing, n = number of orthologues in comparison. A full set of BUSCO scores is available at https://blobtoolkit.genomehubs.org/view/ilEudLacu3.1/dataset/CANOAT01/busco.

**Figure 3.**
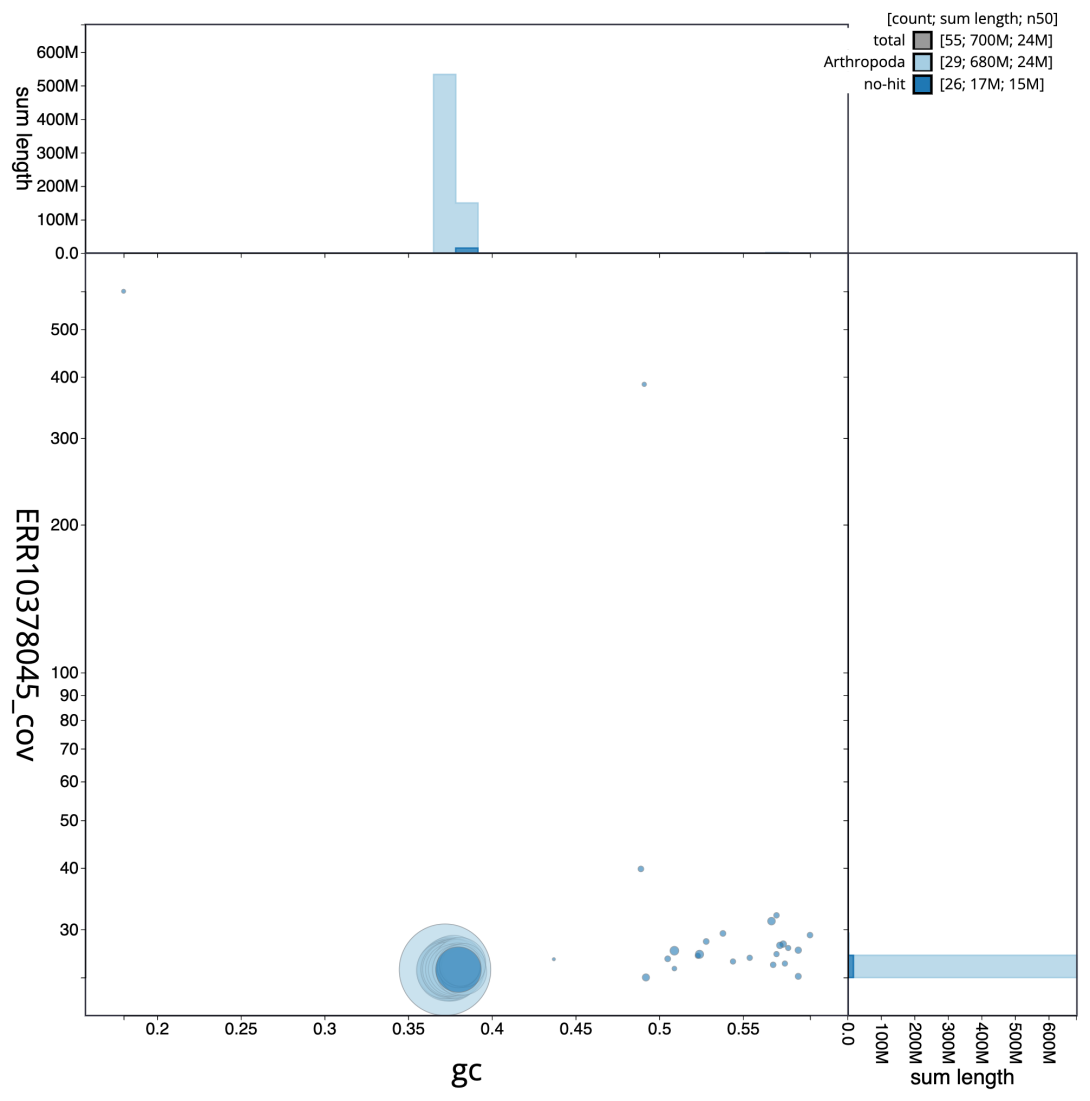
Genome assembly of *Eudonia lacustrata*, ilEudLacu3.1: BlobToolKit GC-coverage plot. Scaffolds are coloured by phylum. Circles are sized in proportion to scaffold length. Histograms show the distribution of scaffold length sum along each axis. An interactive version of this figure is available at https://blobtoolkit.genomehubs.org/view/ilEudLacu3.1/dataset/CANOAT01/blob.

**Figure 4.**
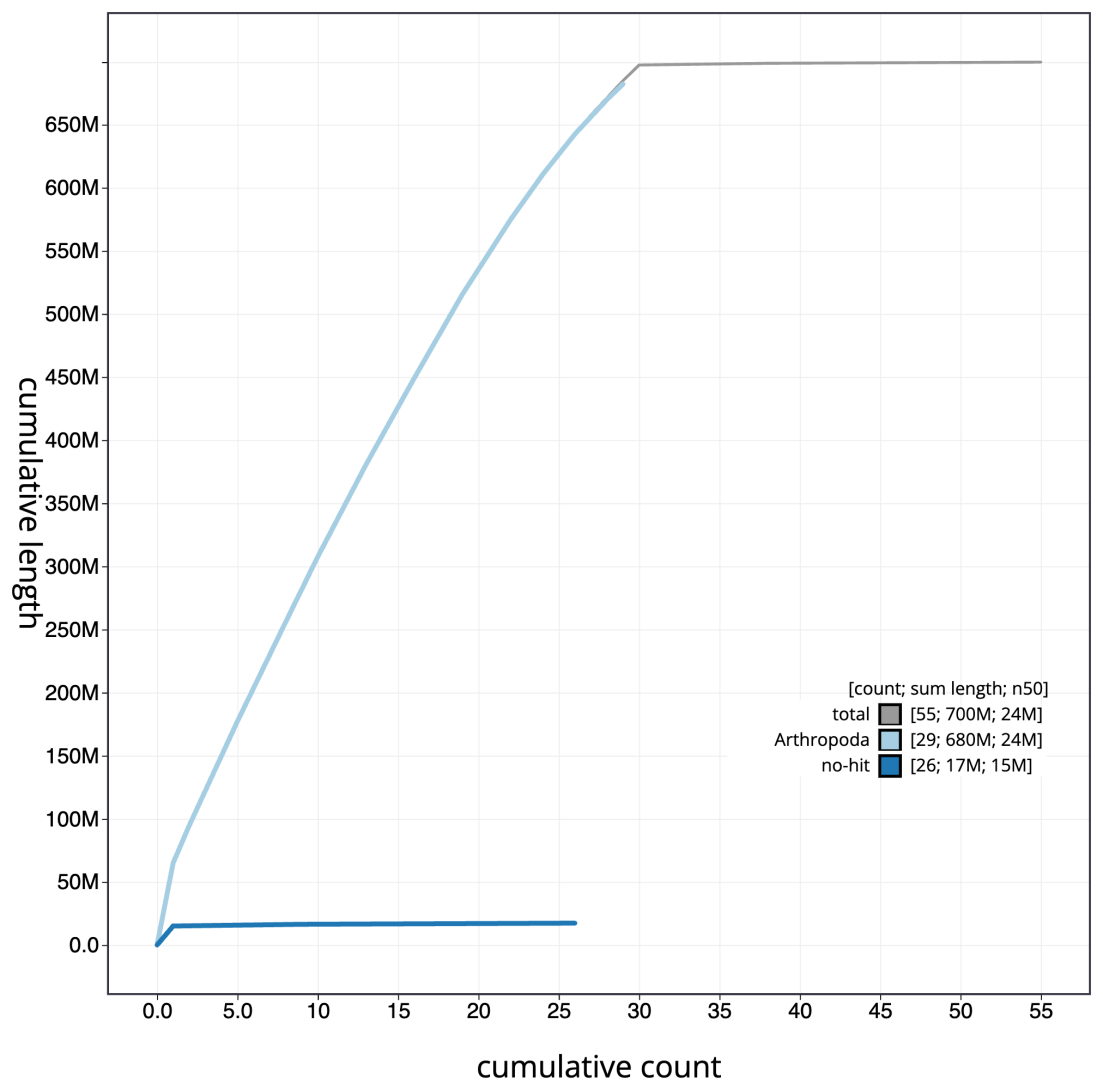
Genome assembly of *Eudonia lacustrata*, ilEudLacu3.1: BlobToolKit cumulative sequence plot. The grey line shows cumulative length for all scaffolds. Coloured lines show cumulative lengths of scaffolds assigned to each phylum using the buscogenes taxrule. An interactive version of this figure is available at https://blobtoolkit.genomehubs.org/view/ilEudLacu3.1/dataset/CANOAT01/cumulative.

**Figure 5.**
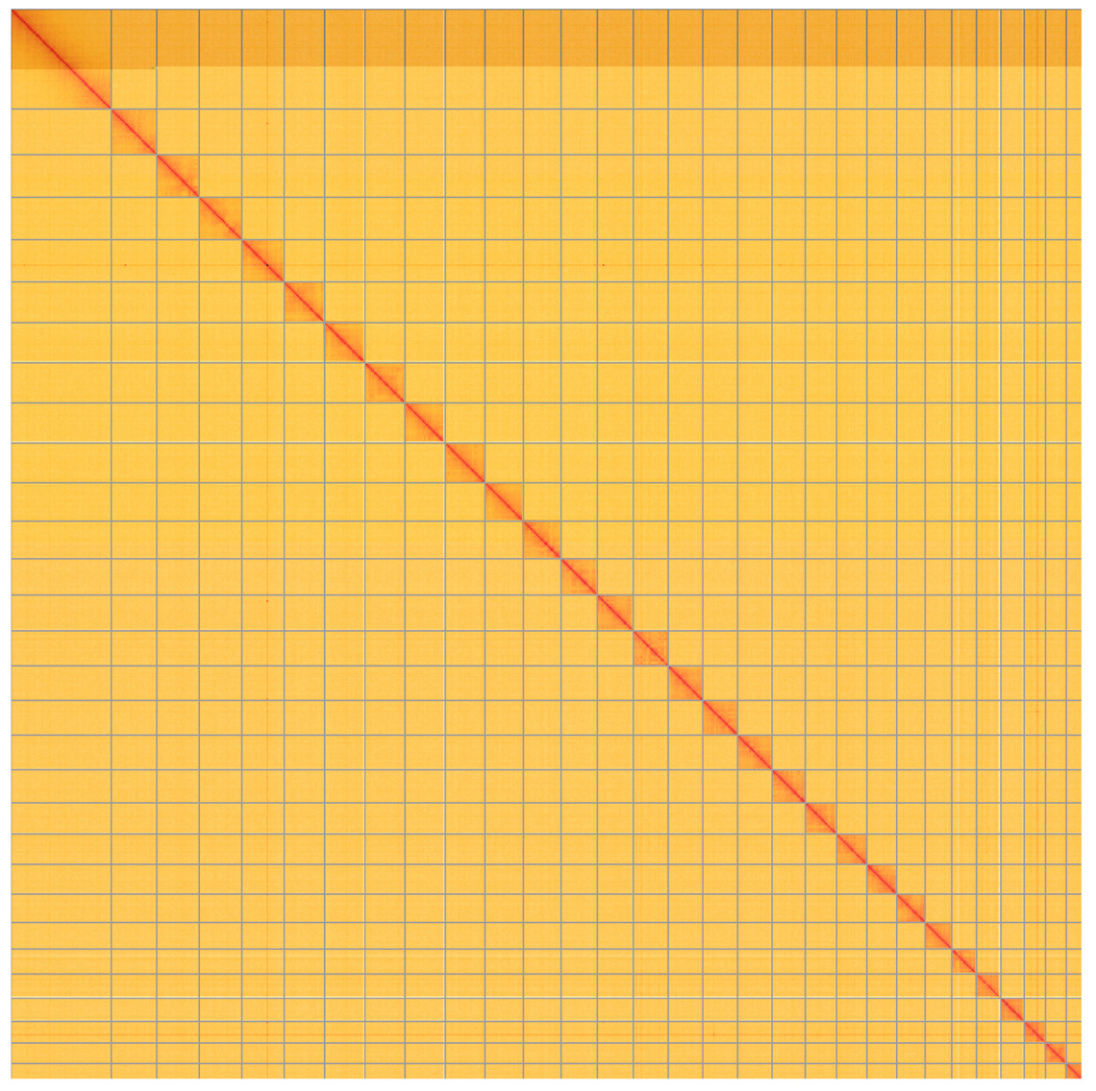
Genome assembly of *Eudonia lacustrata*, ilEudLacu3.1: Hi-C contact map of the ilEudLacu3.1 assembly, visualised using HiGlass. Chromosomes are shown in order of size from left to right and top to bottom. An interactive version of this figure may be viewed at https://genome-note-higlass.tol.sanger.ac.uk/l/?d=Sep94sigTu2RzJYWR6TH2A.

The estimated Quality Value (QV) of the final assembly is 60.6 with *k*-mer completeness of 100%, and the assembly has a BUSCO v5.3.2 completeness of 98.6% (single = 98.0%, duplicated = 0.7%), using the lepidoptera_odb10 reference set ( *n* = 5,286).

Metadata for specimens, spectral estimates, sequencing runs, contaminants and pre-curation assembly statistics can be found at https://links.tol.sanger.ac.uk/species/1100991.

**Table 2.** Chromosomal pseudomolecules in the genome assembly of *Eudonia lacustrata*, ilEudLacu3.

INSDC accession	Chromosome	Length (Mb)	GC%
OX387340.1	1	29.61	37.5
OX387341.1	2	27.62	37.5
OX387342.1	3	27.57	37.5
OX387312.1	4	27.41	37.5
OX387313.1	5	26.35	37.5
OX387314.1	6	26.16	37.5
OX387315.1	7	26.11	37.5
OX387316.1	8	25.96	37.5
OX387317.1	9	25.81	37.5
OX387318.1	10	24.93	37.5
OX387319.1	11	24.46	37.0
OX387320.1	12	23.53	37.5
OX387321.1	13	23.32	37.5
OX387322.1	14	22.61	38.0
OX387323.1	15	22.57	37.5
OX387324.1	16	22.49	37.5
OX387325.1	17	22.38	37.5
OX387326.1	18	21.6	38.0
OX387327.1	19	20.26	37.5
OX387328.1	20	19.58	38.0
OX387329.1	21	19.35	38.0
OX387330.1	22	18.47	37.5
OX387331.1	23	17.33	38.0
OX387332.1	24	16.16	38.0
OX387333.1	25	15.8	38.5
OX387334.1	26	15.05	38.0
OX387335.1	27	13.96	38.5
OX387336.1	28	13.39	38.0
OX387337.1	29	12.35	38.0
OX387339.1	Z	65.03	37.0
OX387338.1	MT	0.02	18.5

## Genome annotation report

The *Eudonia lacustrata* genome assembly ( GCA_947562085.1) was annotated using the Ensembl rapid annotation pipeline ( Table 1). The resulting annotation includes 21,845 transcribed mRNAs from 21,652 protein-coding genes.

## Methods

### Sample acquisition and nucleic acid extraction

Two *Eudonia lacustrata* (specimen IDs Ox0576 and Ox0577) were collected from Wytham Woods, Oxfordshire (biological vice-county Berkshire), UK (latitude 51.77, longitude –1.34) on 2020-07-05, using a light trap. Douglas Boyes (University of Oxford) collected and identified the specimens. The specimens were snap-frozen on dry ice. Specimen Ox0577 (ToLID ilEudLacu3) was used for DNA sequencing and specimen Ox0576 (ToLID ilEudLacu4) was used for Hi-C scaffolding.

A specimen (specimen ID Ox002244, individual ilEudLacu5), collected from the same location on 2022-06-21 by Liam Crowley (University of Oxford), was used for RNA sequencing.

DNA was extracted at the Tree of Life laboratory, Wellcome Sanger Institute (WSI). The ilEudLacu3 sample was weighed and dissected on dry ice with tissue set aside for Hi-C sequencing. Whole organism tissue was disrupted using a Nippi Powermasher fitted with a BioMasher pestle. High molecular weight (HMW) DNA was extracted using the Qiagen MagAttract HMW DNA extraction kit. Low molecular weight DNA was removed from a 20-ng aliquot of extracted DNA using the 0.8X AMpure XP purification kit prior to 10X Chromium sequencing; a minimum of 50 ng DNA was submitted for 10X sequencing. HMW DNA was sheared into an average fragment size of 12–20 kb in a Megaruptor 3 system with speed setting 30. Sheared DNA was purified by solid-phase reversible immobilisation using AMPure PB beads with a 1.8X ratio of beads to sample to remove the shorter fragments and concentrate the DNA sample. The concentration of the sheared and purified DNA was assessed using a Nanodrop spectrophotometer and Qubit Fluorometer and Qubit dsDNA High Sensitivity Assay kit. Fragment size distribution was evaluated by running the sample on the FemtoPulse system.

RNA was extracted from whole organism tissue of ilEudLacu5 in the Tree of Life Laboratory at the WSI using TRIzol, according to the manufacturer’s instructions. RNA was then eluted in 50 μl RNAse-free water and its concentration assessed using a Nanodrop spectrophotometer and Qubit Fluorometer using the Qubit RNA Broad-Range (BR) Assay kit. Analysis of the integrity of the RNA was done using Agilent RNA 6000 Pico Kit and Eukaryotic Total RNA assay.

### Sequencing

Pacific Biosciences HiFi circular consensus and 10X Genomics read cloud DNA sequencing libraries were constructed according to the manufacturers’ instructions. Poly(A) RNA-Seq libraries were constructed using the NEB Ultra II RNA Library Prep kit. DNA and RNA sequencing was performed by the Scientific Operations core at the WSI on Pacific Biosciences SEQUEL II (HiFi), Illumina NovaSeq 6000 (RNA-Seq) and HiSeq X Ten (10X) instruments. Hi-C data were also generated from whole organism tissue of ilEudLacu4 using the Arimav2 kit and sequenced on the Illumina NovaSeq 6000 instrument.

### Genome assembly, curation and evaluation

Assembly was carried out with Hifiasm ( Cheng *et al.*, 2021) and haplotypic duplication was identified and removed with purge_dups ( Guan *et al.*, 2020). One round of polishing was performed by aligning 10X Genomics read data to the assembly with Long Ranger ALIGN, calling variants with FreeBayes ( Garrison & Marth, 2012). The assembly was then scaffolded with Hi-C data ( Rao *et al.*, 2014) using YaHS. The assembly was checked for contamination and corrected as described previously ( Howe *et al.*, 2021). Manual curation was performed using HiGlass ( Kerpedjiev *et al.*, 2018) and Pretext ( Harry, 2022). The mitochondrial genome was assembled using MitoHiFi ( Uliano-Silva *et al.*, 2022), which runs MitoFinder ( Allio *et al.*, 2020) or MITOS ( Bernt *et al.*, 2013) and uses these annotations to select the final mitochondrial contig and to ensure the general quality of the sequence.

A Hi-C map for the final assembly was produced using bwa-mem2 ( Vasimuddin *et al.*, 2019) in the Cooler file format ( Abdennur & Mirny, 2020). To assess the assembly metrics, the *k*-mer completeness and QV consensus quality values were calculated in Merqury ( Rhie *et al.*, 2020). This work was done using Nextflow ( Di Tommaso *et al.*, 2017) DSL2 pipelines “sanger-tol/readmapping” ( Surana *et al.*, 2023a) and “sanger-tol/genomenote” ( Surana *et al.*, 2023b). The genome was analysed within the BlobToolKit environment ( Challis *et al.*, 2020) and BUSCO scores ( Manni *et al.*, 2021; Simão *et al.*, 2015) were calculated.

Table 3 contains a list of relevant software tool versions and sources.

**Table 3.** Software tools: versions and sources.

Software tool	Version	Source
BlobToolKit	4.0.7	https://github.com/blobtoolkit/ https://github.com/blobtoolkit/">blobtoolkit
BUSCO	5.3.2	https://gitlab.com/ezlab/busco
FreeBayes	1.3.1-17-gaa2ace8	https://github.com/freebayes/ https://github.com/freebayes/">freebayes
Hifiasm	0.16.1-r375	https://github.com/chhylp123/ https://github.com/chhylp123/">hifiasm
HiGlass	1.11.6	https://github.com/higlass/ https://github.com/higlass/">higlass
Long Ranger ALIGN	2.2.2	https://support.10xgenomics. com/genome-exome/software/ pipelines/latest/advanced/other- pipelines
Merqury	MerquryFK	https://github.com/ thegenemyers/MERQURY.FK
MitoHiFi	2	https://github.com/ marcelauliano/MitoHiFi
PretextView	0.2	https://github.com/wtsi-hpag/ PretextView
purge_dups	1.2.3	https://github.com/dfguan/ https://github.com/dfguan/">purge_dups
sanger-tol/ genomenote	v1.0	https://github.com/sanger-tol/ genomenote
sanger-tol/ readmapping	1.1.0	https://github.com/sanger-tol/ readmapping/tree/1.1.0
YaHS	yahs- 1.1.91eebc2	https://github.com/c-zhou/yahs

### Genome annotation

The BRAKER2 pipeline ( Brůna *et al.*, 2021) was used in the default protein mode to generate annotation for the *Eudonia lacustrata* assembly (GCA_947562085.1) in Ensembl Rapid Release.

### Wellcome Sanger Institute – Legal and Governance

The materials that have contributed to this genome note have been supplied by a Darwin Tree of Life Partner. The submission of materials by a Darwin Tree of Life Partner is subject to the **‘Darwin Tree of Life Project Sampling Code of Practice’**, which can be found in full on the Darwin Tree of Life website here. By agreeing with and signing up to the Sampling Code of Practice, the Darwin Tree of Life Partner agrees they will meet the legal and ethical requirements and standards set out within this document in respect of all samples acquired for, and supplied to, the Darwin Tree of Life Project.

Further, the Wellcome Sanger Institute employs a process whereby due diligence is carried out proportionate to the nature of the materials themselves, and the circumstances under which they have been/are to be collected and provided for use. The purpose of this is to address and mitigate any potential legal and/or ethical implications of receipt and use of the materials as part of the research project, and to ensure that in doing so we align with best practice wherever possible.

The overarching areas of consideration are: 

•   Ethical review of provenance and sourcing of the material

•   Legality of collection, transfer and use (national and international) 

Each transfer of samples is further undertaken according to a Research Collaboration Agreement or Material Transfer Agreement entered into by the Darwin Tree of Life Partner, Genome Research Limited (operating as the Wellcome Sanger Institute), and in some circumstances other Darwin Tree of Life collaborators.

## Data Availability

European Nucleotide Archive: *Eudonia lacustrata* (little grey). Accession number PRJEB56735; https://identifiers.org/ena.embl/PRJEB56735 ( Wellcome Sanger Institute, 2022) The genome sequence is released openly for reuse. The *Eudonia lacustrata* genome sequencing initiative is part of the Darwin Tree of Life (DToL) project. All raw sequence data and the assembly have been deposited in INSDC databases. Raw data and assembly accession identifiers are reported in Table 1.

## Author information

Members of the University of Oxford and Wytham Woods Genome Acquisition Lab are listed here: https://doi.org/10.5281/zenodo.4789928.

Members of the Darwin Tree of Life Barcoding collective are listed here: https://doi.org/10.5281/zenodo.4893703.

Members of the Wellcome Sanger Institute Tree of Life programme are listed here: https://doi.org/10.5281/zenodo.4783585.

Members of Wellcome Sanger Institute Scientific Operations: DNA Pipelines collective are listed here: https://doi.org/10.5281/zenodo.4790455.

Members of the Tree of Life Core Informatics collective are listed here: https://doi.org/10.5281/zenodo.5013541.

Members of the Darwin Tree of Life Consortium are listed here: https://doi.org/10.5281/zenodo.4783558.

## References

[ref-1] Abdennur N Mirny LA : Cooler: Scalable storage for Hi-C data and other genomically labeled arrays. *Bioinformatics.* 2020;36(1):311–316. 10.1093/bioinformatics/btz540 31290943 PMC8205516

[ref-2] Allio R Schomaker‐Bastos A Romiguier J : MitoFinder: Efficient automated large‐scale extraction of mitogenomic data in target enrichment phylogenomics. *Mol Ecol Resour.* 2020;20(4):892–905. 10.1111/1755-0998.13160 32243090 PMC7497042

[ref-3] Bernt M Donath A Jühling F : MITOS: Improved *de novo* metazoan mitochondrial genome annotation. *Mol Phylogenet Evol.* 2013;69(2):313–319. 10.1016/j.ympev.2012.08.023 22982435

[ref-4] Brůna T Hoff KJ Lomsadze A : BRAKER2: Automatic eukaryotic genome annotation with GeneMark-EP+ and AUGUSTUS supported by a protein database. *NAR Genom Bioinform.* 2021;3(1): lqaa108. 10.1093/nargab/lqaa108 33575650 PMC7787252

[ref-5] Challis R Richards E Rajan J : BlobToolKit – interactive quality assessment of genome assemblies. *G3 (Bethesda).* 2020;10(4):1361–1374. 10.1534/g3.119.400908 32071071 PMC7144090

[ref-6] Cheng H Concepcion GT Feng X : Haplotype-resolved *de novo* assembly using phased assembly graphs with hifiasm. *Nat Methods.* 2021;18(2):170–175. 10.1038/s41592-020-01056-5 33526886 PMC7961889

[ref-7] Di Tommaso P Chatzou M Floden EW : Nextflow enables reproducible computational workflows. *Nat Biotechnol.* 2017;35(4):316–319. 10.1038/nbt.3820 28398311

[ref-8] Garrison E Marth G : Haplotype-based variant detection from short-read sequencing. 2012. 10.48550/arXiv.1207.3907

[ref-9] Guan D McCarthy SA Wood J : Identifying and removing haplotypic duplication in primary genome assemblies. *Bioinformatics.* 2020;36(9):2896–2898. 10.1093/bioinformatics/btaa025 31971576 PMC7203741

[ref-10] Harry E : PretextView (Paired REad TEXTure Viewer): A desktop application for viewing pretext contact maps. 2022; [Accessed 19 October 2022]. Reference Source

[ref-11] Howe K Chow W Collins J : Significantly improving the quality of genome assemblies through curation. *GigaScience.* Oxford University Press,2021;10(1): giaa15. 10.1093/gigascience/giaa153 33420778 PMC7794651

[ref-12] Kerpedjiev P Abdennur N Lekschas F : HiGlass: web-based visual exploration and analysis of genome interaction maps. *Genome Biol.* 2018;19(1):125. 10.1186/s13059-018-1486-1 30143029 PMC6109259

[ref-13] Leraut P : Moths of Europe - Zygaenids, Pyralids 1.N.A.P. Editions,2012;3.

[ref-14] Manni M Berkeley MR Seppey M : BUSCO update: Novel and streamlined workflows along with broader and deeper phylogenetic coverage for scoring of eukaryotic, prokaryotic, and viral genomes. *Mol Biol Evol.* 2021;38(10):4647–4654. 10.1093/molbev/msab199 34320186 PMC8476166

[ref-15] Rao SSP Huntley MH Durand NC : A 3D map of the human genome at kilobase resolution reveals principles of chromatin looping. *Cell.* 2014;159(7):1665–1680. 10.1016/j.cell.2014.11.021 25497547 PMC5635824

[ref-16] Rhie A McCarthy SA Fedrigo O : Towards complete and error-free genome assemblies of all vertebrate species. *Nature.* 2021;592(7856):737–746. 10.1038/s41586-021-03451-0 33911273 PMC8081667

[ref-17] Rhie A Walenz BP Koren S : Merqury: Reference-free quality, completeness, and phasing assessment for genome assemblies. *Genome Biol.* 2020;21(1):245. 10.1186/s13059-020-02134-9 32928274 PMC7488777

[ref-18] Simão FA Waterhouse RM Ioannidis P : BUSCO: assessing genome assembly and annotation completeness with single-copy orthologs. *Bioinformatics.* 2015;31(19):3210–3212. 10.1093/bioinformatics/btv351 26059717

[ref-19] Sterling P Parsons M : Field Guide to the Micro-moths of Great Britain and Ireland.London: Bloomsbury,2018.

[ref-20] Surana P Muffato M Qi G : sanger-tol/readmapping: sanger-tol/readmapping v1.1.0 - Hebridean Black (1.1.0). *Zenodo.* 2023a. 10.5281/zenodo.7755665

[ref-21] Surana P Muffato M Sadasivan Baby C : sanger-tol/genomenote (v1.0.dev). *Zenodo.* 2023b. 10.5281/zenodo.6785935

[ref-22] Uliano-Silva M Ferreira GJRN Krasheninnikova K : MitoHiFi: a python pipeline for mitochondrial genome assembly from PacBio High Fidelity reads. *BioRxiv.* 2022. 10.1101/2022.12.23.521667 PMC10354987 37464285

[ref-23] Vasimuddin M Misra S Li H : Efficient Architecture-Aware Acceleration of BWA-MEM for Multicore Systems.In: *2019 IEEE International Parallel and Distributed Processing Symposium (IPDPS).*IEEE,2019;314–324. 10.1109/IPDPS.2019.00041

[ref-24] Wellcome Sanger Institute: The genome sequence of the Little Grey, *Eudonia lacustrata* (Panzer, 1804). European Nucleotide Archive. [dataset], accession number PRJEB56735,2022.

