# Supplementary material for: The genome sequence of the Little Grey, Eudonia lacustrata (Panzer, 1804)
Source: Wellcome Open Res. 2023 Jul 11;8:302. [Version 1] doi: 10.12688/wellcomeopenres.19646.1 (PMC11255550; doi:10.12688/wellcomeopenres.19646.1)
Supplement: Peer review history

# Reviewer report: Reviewer response for version 1

**DOI:** 10.21956/wellcomeopenres.21763.r87988

**Published:** 2024-07-17

**Authors:** Jerome H L Hui

**Affiliations:** 1 The Chinese University of Hong Kong, Hong Kong, Hong Kong

In this Data Note, Boyes and colleagues sequenced and assembled the genome of a male crambid moth *Eudonia lacustrata* (Panzer, 1804), or commonly known as little grey. According to the NatureSpot, this species has a distribution over most of Great Britain. Molecular data of this species are limited to mitochondrial cytochrome oxidase subunit 1 (COI) sequences and a few other gene sequences deposited to the NCBI database. Therefore, this new genome resource will be useful for further studies, ranging from revealing their population structures, revealing the climate change impact on them, to understanding their evolution with other insects.

 This genome resource is excellent from the summary statistics, with high BUSCO scores, high sequence continuity (scaffold N50), and majority of sequences contained on the 29 pseudochromosomes (plus sex chromosome and mitochondrion). To sum up, this is another valuable contribution from the Darwin Tree of Life Consortium.

Are sufficient details of methods and materials provided to allow replication by others?

Yes

Is the rationale for creating the dataset(s) clearly described?

Yes

Are the datasets clearly presented in a useable and accessible format?

Yes

Are the protocols appropriate and is the work technically sound?

Yes

Reviewer Expertise:

Genomics, evolution, invertebrates

I confirm that I have read this submission and believe that I have an appropriate level of expertise to confirm that it is of an acceptable scientific standard.

# Reviewer report: Reviewer response for version 1

**DOI:** 10.21956/wellcomeopenres.21763.r85350

**Published:** 2024-06-08

**Authors:** Martijn Derks

**Affiliations:** 1 Wageningen University & Research, Wageningen, Gelderland, The Netherlands

This is a well described genome paper. All details are provided and the methodologies are sound. Hence, this genome paper can be indexed. One thing that surprises me a little bit is the number of 21,652 protein-coding genes. This seems to be more than other Lepidoptera? Maybe the authors can comment on that?

Are sufficient details of methods and materials provided to allow replication by others?

Yes

Is the rationale for creating the dataset(s) clearly described?

Yes

Are the datasets clearly presented in a useable and accessible format?

Yes

Are the protocols appropriate and is the work technically sound?

Yes

Reviewer Expertise:

Bioinformatics, Animal Genomics

I confirm that I have read this submission and believe that I have an appropriate level of expertise to confirm that it is of an acceptable scientific standard.
